# Extraintestinal Manifestations and Family History of Inflammatory Bowel Disease Increase the Risk of Pouchitis in a State-Level Epidemiology Study

**DOI:** 10.14309/ctg.0000000000000670

**Published:** 2023-12-26

**Authors:** Edward L. Barnes, Michael S. Dunn, Jean Ashburn, Amy Barto, Richard Bloomfeld, Ashley Cairns, Kurren Mehta, Pooja Patel, Jennifer Dziwis, Scott Esckilsen, Reza Rahbar, Timothy S. Sadiq, John S. Hanson, Hans H. Herfarth

**Affiliations:** 1 Division of Gastroenterology and Hepatology, University of North Carolina, Chapel Hill, North Carolina, USA;; 2 Center for Gastrointestinal Biology and Disease, University of North Carolina, Chapel Hill, North Carolina, USA;; 3 Department of Medicine, University of North Carolina, Chapel Hill, North Carolina, USA;; 4 Division of Colon and Rectal Surgery, Atrium Health at Wake Forest Baptist, Winston-Salem, North Carolina, USA;; 5 Division of Gastroenterology and Hepatology, Duke University, Durham, North Carolina, USA;; 6 Section of Gastroenterology, Atrium Health at Wake Forest Baptist, Winston-Salem, North Carolina, USA;; 7 Department of Medicine, Duke University, Durham, North Carolina, USA;; 8 University of North Carolina School of Medicine, Chapel Hill, North Carolina, USA;; 9 North Carolina Surgery, Raleigh, North Carolina, USA;; 10 Division of Gastroenterology and Hepatology, Atrium Health, Charlotte, North Carolina, USA.

**Keywords:** pouchitis, ileal pouch-anal anastomosis, J-pouch, chronic pouchitis, antibiotics

## Abstract

**INTRODUCTION:**

Our understanding of the epidemiology of inflammatory conditions of the pouch and effectiveness of treatment is largely based on selected populations. We created a state-level registry to evaluate the incidence of pouchitis and the effectiveness of treatments used in an initial episode of pouchitis.

**METHODS:**

In a state-level retrospective cohort of all patients undergoing proctocolectomy with ileal pouch-anal anastomosis (IPAA) for ulcerative colitis between January 1, 2018, and December 31, 2020, we evaluated the incidence of pouchitis and compared the proportion of patients developing recurrent pouchitis and chronic antibiotic-dependent pouchitis according to initial antibiotic therapy.

**RESULTS:**

A total of 177 patients underwent surgery with 49 (28%) developing pouchitis within the 12 months after the final stage of IPAA. Patients with extraintestinal manifestations of inflammatory bowel disease (IBD) were significantly more likely to develop pouchitis within the first 12 months after IPAA (adjusted odds ratio 2.45, 95% confidence interval 1.03–5.81) after adjusting for family history of IBD (adjusted odds ratio 3.50, 95% 1.50–8.18). When comparing the proportion of patients who developed recurrent pouchitis or chronic antibiotic-dependent pouchitis with those who experienced an isolated episode of pouchitis, there were no significant differences among the initial antibiotic regimens used.

**DISCUSSION:**

In a state-level examination of outcomes after IPAA for ulcerative colitis, patients with extraintestinal manifestations of IBD were more likely to develop pouchitis; however, the initial antibiotic regimen chosen did not seem to affect long-term outcomes.

## INTRODUCTION

Pouchitis is the most common complication after ileal pouch-anal anastomosis (IPAA) for ulcerative colitis (UC). Although colectomy has been described as a curative surgery for many patients with UC, in reality, inflammatory complications after IPAA are common with almost one-half of patients developing pouchitis in the first 2 years after IPAA for UC (1) and approximately 1 in 5 patients going on to develop chronic pouchitis (2).

Given indications that the incidence of pouchitis may actually be increasing in recent decades (3), there is a defined need for an improved understanding of the epidemiology and natural history after IPAA for UC. Much of our understanding of the natural history after IPAA for UC has been generated from single-center studies or examinations of selected populations (2,4–10). These studies have provided a foundation for clinical practice; however, questions remain regarding their generalizability to broader populations (11) and implementation. Specifically, given the potential increasing incidence and prevalence of inflammatory conditions of the pouch, improved understanding of early interventions as an opportunity to prevent later complications after IPAA are paramount, including real-world studies of comparative effectiveness.

The North Carolina Pouch Project is a state-level registry that was created for the evaluation of outcomes after successful restorative proctocolectomy with IPAA for UC. Given the unique opportunity to evaluate most patients undergoing IPAA for UC in North Carolina during the study period, the primary objectives of this study were to evaluate both the incidence of pouchitis in the first 12 months after the final stage of IPAA surgery and the treatment strategies used for the initial episode of pouchitis.

## METHODS

### Patient selection

We evaluated 177 patients who underwent IPAA for UC at 1 of 4 tertiary care referral inflammatory bowel disease (IBD) centers in North Carolina between January 1, 2018, and December 31, 2020. The catchment area represented by the 4 centers (Atrium Health Charlotte, Atrium Health Wake Forest Baptist, Duke Health, and University of North Carolina Health) represents the majority of the state of North Carolina. Based on discussions with colorectal surgeons with other large health care systems in North Carolina, it is estimated that less than 10 IPAA surgeries are performed per year outside these 4 centers/health systems.

All study investigators agreed on a standard case report form and strategies for the identification of study subjects before study initiation. Using this standardized case report form, pertinent clinical, demographic, and laboratory data were extracted from the electronic medical record and entered into a central Research Electronic Data Capture data tool, hosted at the University of North Carolina at Chapel Hill.

To be included in the final study population, patients were required to meet the following criteria: (i) age 18 years or older, (ii) documented proctocolectomy with IPAA for UC at 1 of the 4 participating centers or health systems during the study period (with documented preoperative diagnosis of UC), (iii) at least 12 months of follow-up after surgery. Patients younger than 18 years during surgery and those patients with a preoperative diagnosis of Crohn's disease (CD) or IBD unclassified during colectomy were excluded. No patients were excluded because of less than 12 months of follow-up after surgery.

### Outcomes of interest

The primary outcome of interest was the incidence of pouchitis within the first 12 months after restorative proctocolectomy with IPAA for UC. We were specifically interested in the incidence of pouchitis within the first 12 months after IPAA given (A) our interest in practice patterns and treatment effectiveness for the first episode of pouchitis and (B) prior studies indicating that early pouchitis is associated with an increased risk of chronic inflammatory conditions of the pouch (12,13). A diagnosis of pouchitis was based on clinical symptoms including frequency, urgency, abdominal pain, and a sense of malaise, as determined by the evaluating/treating gastroenterologist or colorectal surgeon. For a clinical diagnosis of pouchitis, endoscopic confirmation was not required; however, where endoscopy was available pouchoscopy could be used to confirm the clinical diagnosis because this clinical approach to diagnosis has been used in prior retrospective studies and compared with pouchoscopy confirmation (12,14). We also evaluated the primary treatment strategies used in the management of an initial episode of pouchitis. The efficacy of these treatment strategies was judged in terms of long-term success, evaluating the proportion of patients who experienced recurrent pouchitis and chronic antibiotic-dependent pouchitis (CADP). CADP was defined as pouchitis episodes occurring at least 4 times per year, requiring recurrent courses of antibiotics or continuous antibiotic therapy with symptoms remaining responsive to antibiotic therapy (15,16).

### Covariates

We collected demographic and clinical variables that have been associated with increased complications after IPAA, including pouchitis, such as age, sex, any history of extraintestinal manifestations (EIMs) (17) (including PSC (18–20), arthralgia/arthritis, erythema nodosum, pyoderma gangrenosum, ocular EIMs, and oral aphthae) before surgery, smoking status (21,22), the use of nonsteroidal anti-inflammatory drugs (19,21), the indication for colectomy (23), and stages in IPAA surgery (24). Disease extent of UC during colectomy was also evaluated using the Montreal classification, defining extensive colitis, left-sided colitis, and proctitis as inflammation extending beyond the splenic flexure, distal to the splenic flexure, and affecting the rectum only, respectively (25,26). When evaluating the stages of IPAA surgery, a modified 2-stage IPAA was defined in the following manner: in the first operation, a total abdominal colectomy with end ileostomy is completed; after a recovery interval, a second surgery is performed including completion proctectomy and IPAA without a defunctioning loop ileostomy (14,27).

### Statistical analysis

We used descriptive statistics to summarize demographic and clinical characteristics among patients who developed pouchitis and those who did not. Continuous variables were reported as medians with interquartile range (IQR) while categorical variables were reported as raw values with percentages. Continuous variables were compared using the Wilcoxon rank sum testing given the assumption of non-normal distribution, while categorical variables were compared using the Fisher exact and χ^2^ testing as appropriate. We used multivariable logistic regression to evaluate the odds of developing pouchitis and recurrent/chronic inflammatory conditions of the pouch, adjusting for potential confounders. For all analyses, 2-sided *P* values of 0.05 or less were considered statistically significant. All analyses were performed using SAS version 9.4 (SAS Institute, Cary, NC).

### Ethics

This study was approved by the Institutional Review Board of the University of North Carolina at Chapel Hill and all participating centers.

## RESULTS

### Development of pouchitis and chronic inflammatory conditions of the pouch

Among 177 patients undergoing restorative proctocolectomy with IPAA at 1 of the 4 health centers/systems during the study period, 49 (28%) developed pouchitis within the 12 months after the final stage of IPAA. The median time to development of pouchitis was 211 days (IQR 144–371). There was no significant difference in the proportion of patients developing pouchitis when comparing the 4 health care systems/centers (see Supplementary Table 1, Supplementary Digital Content 1, http://links.lww.com/CTG/B56). The median age during the final stage of IPAA surgery was 39.9 years (IQR 28.9–54.7) and with 41% of the population being female.

When comparing patients who developed pouchitis with those who developed no pouchitis, patients who developed pouchitis were more likely to have a family history of IBD (30% vs 11%, *P* = 0.003) and EIM of IBD (37% vs 15%, *P* = 0.001, Table 1). When evaluating PSC specifically, patients who developed pouchitis were more likely to have a history of PSC compared with those patients who did not develop pouchitis (16% vs 3%, *P* = 0.004). In the evaluation of prior medication exposures, patients who developed pouchitis were more likely to have used methotrexate pre colectomy (12% vs 2%, *P* = 0.008) and vedolizumab (33% vs 13%, *P* = 0.004). Specific to methotrexate use, 18% of patients with EIM of IBD before surgery used methotrexate compared with 3% of patients without EIM of IBD (*P* = 0.059). In an additional follow-up assessment of only those patients with EIM of IBD before surgery, patients who developed pouchitis were no more likely to have used methotrexate than those patients who did not develop pouchitis (31% vs 7%, *P* = 0.153); however, this evaluation included only 5 patients.

**Table 1.** Comparison of demographic and clinical characteristics among patients who developed pouchitis with those who did not

	No pouchitis (n = 128)	Pouchitis (n = 49)	*P* value
Age at IPAA, median (IQR)	39.5 (28.9–54.3)	41.5 (27.4–56.3)	0.899
Female sex, n (%)	55 (43)	18 (37)	0.451
Race, n (%)			0.175
White	105 (82)	45 (92)	
Black or African American	13 (10)	1 (2)	
Other	10 (8)	3 (6)	
Hispanic ethnicity, n (%)	3 (2)	2 (4)	0.532
BMI during IPAA, n (%)			0.700
Underweight	7 (5)	2 (4)	
Normal	57 (15)	23 (47)	
Overweight	37 (29)	17 (35)	
Obese	27 (21)	7 (14)	
Disease extent before surgery, n (%)^a^			0.751
Proctitis	4 (4)	1 (2)	
Left-sided	22 (20)	11 (25)	
Extensive colitis	83 (76)	32 (73)	
Indication for surgery, n (%)			0.397
Medically refractory colitis	98 (77)	41 (85)	
Dysplasia/colorectal cancer	12 (9)	2 (4)	
Other	15 (12)	3 (6)	
Medically refractory disease and dysplasia/colorectal cancer	3 (2)	2 (4)	
No. of stages in surgery, n (%)			0.150
I	6 (5)	0 (0)	
II	35 (27)	8 (16)	
Modified II	43 (34)	21 (43)	
III	44 (34)	20 (41)	
Smoking status during colectomy, n (%)			0.875
Current smoker	3 (2)	1 (2)	
Former smoker	34 (27)	15 (31)	
Never smoker	90 (71)	33 (67)	
Family history of IBD, n (%)	14 (11)	14 (30)	0.003
Primary sclerosing cholangitis, n (%)	4 (3)	8 (16)	0.004
Other extraintestinal manifestations of IBD, n (%)	16 (13)	13 (27)	0.024
Complications within 30 d of IPAA surgery, n (%)	52 (41)	14 (29)	0.138
Pathology findings during colectomy, n (%)			
Granulomas	4 (3)	4 (8)	0.220
Ileitis	22 (17)	5 (10)	0.350
Transmural inflammation	20 (16)	4 (8)	0.229
NSAIDs during IPAA	36 (29)	13 (27)	0.823
Therapy in 3 mo before colectomy, n (%)			
Oral steroids	62 (49)	30 (61)	0.153
Oral aminosalicylate	43 (34)	22 (45)	0.186
Topical aminosalicylate	16 (13)	7 (15)	0.743
Thiopurine (azathioprine or mercaptopurine)	18 (14)	11 (23)	0.172
Methotrexate	3 (2)	6 (12)	0.008
Tofacitinib	19 (15)	7 (14)	1.000
Anti-TNF monotherapy	51 (40)	21 (43)	0.774
Ustekinumab	6 (5)	6 (12)	0.079
Vedolizumab	17 (13)	16 (33)	0.004

BMI, body mass index; IBD, inflammatory bowel disease; IPAA, ileal pouch-anal anastomosis; IQR, interquartile range; NSAID, nonsteroidal anti-inflammatory drug; TNF, tumor necrosis factor.

a Disease extent at the time of surgery unknown for 24 patients.

In a multivariable logistic regression, patients with EIM of IBD demonstrated a significant increase in the odds of developing pouchitis within the first 12 months after IPAA (adjusted odds ratio 2.45, 95% confidence interval 1.03–5.81) after adjusting for family history of IBD (adjusted odds ratio 3.50, 95% 1.50–8.18, Table 2). There were no significant associations between clinical factors and subsequent development of chronic pouchitis. Only 7 (4%) patients developed Crohn's-like disease of the pouch during the study period.

**Table 2.** Odds of developing pouchitis after ileal pouch-anal anastomosis for ulcerative colitis

	Unadjusted analysis		Adjusted analysis	
	OR	95% CI	OR	95% CI
Extraintestinal manifestations of IBD	3.33	1.56–7.11	2.45	1.03–5.81
Family history of IBD	3.45	1.50–7.97	3.50	1.50–8.18

CI, confidence interval; IBD, inflammatory bowel disease; OR, odds ratio.

### Initial antibiotic choices

For the 49 patients treated for an initial episode of pouchitis, 31 (63%) received a fluoroquinolone monotherapy, 3 (6%) received metronidazole monotherapy, 6 (12%) received an alternative antibiotic (amoxicillin-clavulanate or rifaximin), and 9 (18%) received a combination of fluoroquinolone and metronidazole. Although the numbers were small for individual categories, there seemed to be variability in prescribing patterns based on individual sites when analyzing by antibiotic used (see Supplementary Table 2, Supplementary Digital Content 1, http://links.lww.com/CTG/B56) and the duration of initial antibiotic course (see Supplementary Table 3, Supplementary Digital Content 1, http://links.lww.com/CTG/B56). When comparing the proportion of patients who developed recurrent pouchitis with those who experienced an isolated episode of pouchitis, there were no significant differences among the initial antibiotic regimens used (Table 3). Similarly, the initial antibiotic choice did not seem to affect the likelihood of development of CADP.

**Table 3.** Initial antibiotic choices for patients with pouchitis

	No recurrent pouchitis (n = 14), n (%)	Recurrent pouchitis (n = 35), n (%)	*P* value
Fluoroquinolone	7 (23)	24 (77)	0.240
Metronidazole	0 (0)	3 (100)	
Amoxicillin-clavulanate or rifaximin	3 (50)	3 (50)	
Combination fluoroquinolone and metronidazole	4 (44)	5 (56)	

	No chronic antibiotic-dependent pouchitis (n = 26), n (%)	Chronic antibiotic-dependent pouchitis (n = 23), n (%)	*P* value
Fluoroquinolone	15 (48)	16 (52)	0.396
Metronidazole	1 (33)	2 (67)	
Amoxicillin-clavulanate or rifaximin	3 (50)	3 (50)	
Combination fluoroquinolone and metronidazole	7 (78)	2 (22)	

Among 49 patients developing pouchitis, 14 (29%) used an antibiotic course of >14 days for the initial treatment duration. There was no significant difference in the subsequent development of recurrent pouchitis or CADP when comparing patients who were treated with ≤14 days of antibiotics with those treated with >14 days (recurrent pouchitis: 71% vs 71%, *P* = 1.00; CADP: 37% vs 65%, *P* = 0.116).

## DISCUSSION

In this state-level analysis of the epidemiology and natural history after IPAA, we provide a new method of evaluating the incidence of pouchitis in the first year after IPAA for UC and the treatment patterns used for an initial episode of pouchitis. Given a lack of society guidelines to standardize best practices in the treatment of pouchitis and a paucity of comparative effectiveness studies, understanding the real-world effectiveness of therapies used in the treatment of pouchitis and variability in practice and any downstream effects of this variability is paramount. By studying, essentially each patient undergoing IPAA in our state during the study period, we had the ability to evaluate not only potential risk factors of pouchitis but also the influence of immediate treatment on subsequent episodes of pouchitis, suggesting that in the current era, patient and provider preference may guide initial treatment decisions.

Recent studies have indicated that the incidence of pouchitis is increasing in recent years (3), perhaps due to increased disease severity or worsened prognosis during colectomy (28) including the need for and failure of advanced therapies with biologics or small molecules (1,29,30). Understanding the risk of pouchitis, and particularly any novel actionable risk factors of inflammatory conditions of the pouch, remains a critical goal in the current era (31). Despite the unique data source as a state-level epidemiologic study, the sample size represented by the North Carolina Pouch Project is actually smaller than many prior evaluations by single, highly specialized referral centers that have studied both the epidemiology of pouchitis and potential risk factors (2,6,12,14,29,32,33). Future studies may emphasize leveraging both clinical variables and emerging novel methods of risk stratification, and assessment such as multiomic approaches (34–37) may improve our ability to identify those patients at the highest risk of inflammatory conditions of the pouch.

Patients with EIM of IBD demonstrated a significant increase in the odds of developing pouchitis. This association has previously been demonstrated in a large systematic review and meta-analysis (17). In our analysis, only 12 patients had PSC, with patients with PSC being statistically more likely to develop pouchitis than patients with UC alone; however, the small sample size prevented further analysis of this relationship. In prior population-based studies and a recent meta-analysis, patients with PSC have demonstrated a markedly increased risk of the development of pouchitis (1,38,39) leading some authors to argue that PSC-related pouch inflammation represents a unique phenotype (40). Given the strong association between PSC and pouchitis (and chronic pouchitis and pouch failure) (38), patients with PSC may represent a high-risk population that would lead to early intervention for the primary or secondary prevention of inflammatory conditions of the pouch in future studies.

Recent studies have demonstrated the profound shifts that occur in the microbiome among patients treated with antibiotics for pouchitis, thus understanding the role of antibiotic exposures on both initial treatment success and long-term outcomes including the development of recurrent pouchitis, and potentially CADP is of paramount importance. In a study of 49 patients who had undergone IPAA, those patients treated with antibiotics for pouchitis demonstrated multiple ciprofloxacin-resistance mutations in drug target genes and an abrupt shift in microbiome composition after antibiotic cessation (41). These findings detailed by Dubinsky and colleagues led the authors to consider practices including purposeful antibiotic use to minimize chronic inflammation and antibiotic dependence. In our study, there were no significant differences in the proportion of patients developing recurrent pouchitis when evaluating the antibiotic chosen for an initial episode of pouchitis. Although there is a paucity of studies evaluating the comparative efficacy of antibiotic regimens for pouchitis, ciprofloxacin and metronidazole are the generally accepted first-line antibiotic therapies for pouchitis (15,42). One randomized controlled trial suggested that ciprofloxacin may be better tolerated than metronidazole (43), and when reviewing practice patterns across the cohort, most of the patients were treated with a fluoroquinolone monotherapy or combination therapy (fluoroquinolone and metronidazole) when compared with metronidazole monotherapy. However, when comparing long-term outcomes, there was no difference in the prevention of recurrent pouchitis when comparing the initial antibiotic chosen. In this cohort study, we did not evaluate short-term outcomes such as therapy discontinuation or adverse events related to the initial antibiotic therapy chosen.

This study has several strengths including using novel infrastructure to report outcomes using a state-level epidemiology database after IPAA for UC; however, there are limitations. The 4 centers or health care systems represented in this cohort are tertiary care referral centers, and although these effectively represent all patients in the state of North Carolina undergoing IPAA during the study period, there may be an underlying bias when evaluating long-term outcomes such as pouchitis. The initial treatment choice was per the discretion of the treating physician, and we were unable to document the rationale for choosing one antibiotic (or duration) over another. Given that this was a retrospective study, we also do not have corresponding microbiome data during initial diagnosis of pouchitis or in longitudinal follow-up to assess changes in microbial profiles among patients treated with antibiotics. This remains a goal for future prospective research studies. Although this was designed as a state-level database and other colorectal surgeons in the state of North Carolina were queried regarding IPAA volume, we recognize that some patients undergoing IPAA surgery in our state are not represented as estimated in the methods. Finally, we relied on a clinical diagnosis of pouchitis given that this was the first episode of pouchitis and pouchoscopy is not universally recommended for an initial presentation with classic symptoms of pouchitis (44). Prior work in retrospective populations has also demonstrated strong correlation between clinical symptoms and pouchoscopy findings in initial pouchitis presentations (14).

In this state-level analysis of outcomes after IPAA for UC, almost one-third of patients developed pouchitis within the first 12 months after IPAA. The antibiotic chosen for an initial episode of pouchitis did not seem to influence rates of recurrent pouchitis or CADP, indicating that patient preference/tolerance and shared decision-making based on physician assessment should likely guide decision-making for the initial antibiotic choices at this time. Future prospective studies focused on the underlying mechanisms of pouchitis may be more informative in terms of the real-world effectiveness of specific antibiotics in this setting.

## CONFLICTS OF INTEREST

**Guarantor of the article:** Edward L. Barnes, MD, MPH, FACG.

**Specific author contributions:** E.L.B.: designed the study, was responsible for study supervision and data maintenance, performed all data analyses, wrote the first draft of the manuscript, and participated in revising the manuscript. S.E., A.C., K.M., P.P., J.D., and M.S.D.: participated in the study design, were responsible for data acquisition and data maintenance, and revised the manuscript. J.A., A.B., R.B., J.S.H., and H.H.H.: participated in the study design, were responsible for study supervision, and revised the manuscript. R.R. and T.S.S.: participated in the study design, were responsible for study supervision, and revised the manuscript. All authors agree with the final version of the manuscript and are accountable for the work presented.

**Financial support:** This research was supported by grants from the National Institutes of Health (K23DK127157-01) (E.L.B.).

**Potential competing interests:** E.L.B. has served as a consultant for Bristol-Meyers Squibb and Target RWE. H.H.H. has served as a consultant for BMS, ExeGi, Finch, Fresenius Kabi, Galapagos, Gilead, Janssen, Otsuka, Ventyx; and received research support from Allakos, Artizan, NovoNordisk, and Pfizer. The remaining authors have no relevant disclosures.

**Study Highlights**

**WHAT IS KNOWN**

✓ Pouchitis is the most common complication after ileal pouch-anal anastomosis for ulcerative colitis.
✓ Ciprofloxacin and metronidazole are the most commonly used antibiotics for the treatment of pouchitis, but the ideal antibiotic treatment has not been established.

**WHAT IS NEW HERE**

✓ This is a state-level outcomes assessment of patients undergoing ileal pouch-anal anastomosis for ulcerative colitis.
✓ As demonstrated in other studies, patients with extraintestinal manifestations of inflammatory bowel disease were at an increased risk of developing pouchitis along with those with a family history of inflammatory bowel disease.
✓ There was no significant difference in the proportion of patients developing recurrent pouchitis or chronic antibiotic-dependent pouchitis when comparing the antibiotic regimen used to treat an initial episode of pouchitis.

## References

[1] Barnes EL Herfarth HH Kappelman MD . Incidence, risk factors, and outcomes of pouchitis and pouch-related complications in patients with ulcerative colitis. Clin Gastroenterol Hepatol 2021;19(8):1583–91.e4. 32585362 10.1016/j.cgh.2020.06.035 PMC8552292

[2] Fazio VW Kiran RP Remzi FH . Ileal pouch anal anastomosis: Analysis of outcome and quality of life in 3707 patients. Ann Surg 2013;257(4):679–85. 23299522 10.1097/SLA.0b013e31827d99a2

[3] Barnes EL Allin KH Iversen AT . Increasing incidence of pouchitis between 1996 and 2018: A population-based Danish cohort study. Clin Gastroenterol Hepatol 2023;21(1):192–9 e7. 35525393 10.1016/j.cgh.2022.04.015 PMC9636065

[4] Fazio VW Ziv Y Church JM . Ileal pouch-anal anastomoses complications and function in 1005 patients. Ann Surg 1995;222(2):120–7. 7639579 10.1097/00000658-199508000-00003 PMC1234769

[5] Hahnloser D Pemberton JH Wolff BG . Results at up to 20 years after ileal pouch-anal anastomosis for chronic ulcerative colitis. Br J Surg 2007;94(3):333–40. 17225210 10.1002/bjs.5464

[6] Lightner AL Mathis KL Dozois EJ . Results at up to 30 years after ileal pouch-anal anastomosis for chronic ulcerative colitis. Inflamm Bowel Dis 2017;23(5):781–90. 28301429 10.1097/MIB.0000000000001061

[7] Kayal M Plietz M Rizvi A . Inflammatory pouch conditions are common after ileal pouch anal anastomosis in ulcerative colitis patients. Inflamm Bowel Dis 2020;26(7):1079–86. 31587035 10.1093/ibd/izz227 PMC7456971

[8] Datta I Buie WD Maclean AR . Hospital readmission rates after ileal pouch-anal anastomosis. Dis Colon Rectum 2009;52(1):55–8. 19273956 10.1007/DCR.0b013e31819724a3

[9] Ozturk E Kiran RP Remzi F . Early readmission after ileoanal pouch surgery. Dis Colon Rectum 2009;52(11):1848–53. 19966631 10.1007/DCR.0b013e3181b15610

[10] Hanzlik TP Tevis SE Suwanabol PA . Characterizing readmission in ulcerative colitis patients undergoing restorative proctocolectomy. J Gastrointest Surg 2015;19(3):564–9. 25560185 10.1007/s11605-014-2734-7 PMC4565166

[11] McKenna NP Habermann EB Glasgow AE . Risk factors for readmission following ileal pouch-anal anastomosis: An American College of Surgeons National Surgical Quality Improvement Program analysis. J Surg Res 2018;229:324–31. 29937009 10.1016/j.jss.2018.04.037

[12] Esckilsen S Kochar B Weaver KN . Very early pouchitis is associated with an increased likelihood of chronic inflammatory conditions of the pouch. Dig Dis Sci 2023;68(7):3139–47. 37148442 10.1007/s10620-023-07947-9

[13] Kayal M Kohler D Plietz M . Early pouchitis is associated with Crohn's disease-like pouch inflammation in patients with ulcerative colitis. Inflamm Bowel Dis 2022;28(12):1821–5. 35188532 10.1093/ibd/izac012 PMC9924036

[14] Sherrill GC Esckilsen S Hudson J . Relationship between stages of ileal pouch-anal anastomosis, timing of restoration of fecal continuity, and pouchitis. Dig Dis Sci 2022;67(11):5220–6. 35246803 10.1007/s10620-022-07440-9

[15] Barnes EL Lightner AL Regueiro M. Peri-operative and post-operative management of patients with Crohn's disease and ulcerative colitis. Clin Gastroenterol Hepatol 2020;18(6):1356–66. 31589972 10.1016/j.cgh.2019.09.040

[16] Quinn KP Raffals LE. An update on the medical management of inflammatory pouch complications. Am J Gastroenterol 2020;115(9):1439–50. 32453044 10.14309/ajg.0000000000000666

[17] Hata K Okada S Shinagawa T . Meta-analysis of the association of extraintestinal manifestations with the development of pouchitis in patients with ulcerative colitis. BJS Open 2019;3(4):436–44. 31463422 10.1002/bjs5.50149 PMC6706792

[18] Hata K Watanabe T Shinozaki M . Patients with extraintestinal manifestations have a higher risk of developing pouchitis in ulcerative colitis: Multivariate analysis. Scand J Gastroenterol 2003;38(10):1055–8. 14621280 10.1080/00365520310005938

[19] Lepisto A Karkkainen P Jarvinen HJ. Prevalence of primary sclerosing cholangitis in ulcerative colitis patients undergoing proctocolectomy and ileal pouch-anal anastomosis. Inflamm Bowel Dis 2008;14(6):775–9. 18253951 10.1002/ibd.20384

[20] White E Melmed GY Vasiliauskas EA . A prospective analysis of clinical variables, serologic factors, and outcome of ileal pouch-anal anastomosis in patients with backwash ileitis. Dis Colon Rectum 2010;53(7):987–94. 20551749 10.1007/DCR.0b013e3181dcb3f2 PMC5002988

[21] Achkar JP Al-Haddad M Lashner B . Differentiating risk factors for acute and chronic pouchitis. Clin Gastroenterol Hepatol 2005;3(1):60–6. 15645406 10.1016/s1542-3565(04)00604-4

[22] Shen B Fazio VW Remzi FH . Risk factors for diseases of ileal pouch-anal anastomosis after restorative proctocolectomy for ulcerative colitis. Clin Gastroenterol Hepatol 2006;4(1):81–9; quiz 2–3. 16431309 10.1016/j.cgh.2005.10.004

[23] Yanai H Ben-Shachar S Mlynarsky L . The outcome of ulcerative colitis patients undergoing pouch surgery is determined by pre-surgical factors. Aliment Pharmacol Ther 2017;46(5):508–15. 28664992 10.1111/apt.14205

[24] Kochar B Barnes EL Peery AF . Delayed ileal pouch anal anastomosis has a lower 30-day adverse event rate: Analysis from the National Surgical Quality Improvement Program. Inflamm Bowel Dis 2018;24(8):1833–9. 29697787 10.1093/ibd/izy082 PMC6703434

[25] Satsangi J Silverberg MS Vermeire S . The Montreal classification of inflammatory bowel disease: Controversies, consensus, and implications. Gut 2006;55(6):749–53. 16698746 10.1136/gut.2005.082909 PMC1856208

[26] Silverberg M Satsangi J Ahmad T . Toward an integrated clinical, molecular and serological classification of inflammatory bowel disease: Report of a working party of the 2005 Montreal World Congress of Gastroenterology. Can J Gastroenterol 2005;19(Suppl A):5A–36A. 10.1155/2005/269076 16151544

[27] Samples J Evans K Chaumont N . Variant two-stage ileal pouch-anal anastomosis: An innovative and effective alternative to standard resection in ulcerative colitis. J Am Coll Surg 2017;224(4):557–63. 28315811 10.1016/j.jamcollsurg.2016.12.049

[28] Kayal M Posner H Milwidsky HM . Acute severe ulcerative colitis is associated with an increased risk of acute pouchitis. Inflamm Bowel Dis 2023;29(12):1907–11. 36939632 10.1093/ibd/izad039 PMC12102480

[29] Bertucci Zoccali M Hyman NH Skowron KB . Exposure to anti-tumor necrosis factor medications increases the incidence of pouchitis after restorative proctocolectomy in patients with ulcerative colitis. Dis Colon Rectum 2019;62(11):1344–51. 31596761 10.1097/DCR.0000000000001467

[30] Runde J Erondu A Akiyama S . Outcomes of ileoanal pouch anastomosis in pediatric ulcerative colitis are worse in the modern era: A time trend analysis outcomes following ileal pouch-anal anastomosis in pediatric ulcerative colitis. Inflamm Bowel Dis 2022;28(9):1386–94. 35040964 10.1093/ibd/izab319 PMC9434476

[31] Barnes EL Chang S. Shedding new light on high-risk phenotypes for pouchitis. Inflamm Bowel Dis 2023;29(12):2003–5. 36869847 10.1093/ibd/izad027 PMC10697420

[32] Barnes EL Raffals L Long MD . Disease and treatment patterns among patients with pouch-related conditions in a cohort of large tertiary care inflammatory bowel disease centers in the United States. Crohns Colitis 360 2020;2(3):otaa039. 32744536 10.1093/crocol/otaa039 PMC7380550

[33] Fleshner P Ippoliti A Dubinsky M . A prospective multivariate analysis of clinical factors associated with pouchitis after ileal pouch-anal anastomosis. Clin Gastroenterol Hepatol 2007;5(8):952–8; quiz 887. 17544871 10.1016/j.cgh.2007.03.020

[34] Devlin JC Axelrad J Hine AM . Single-cell transcriptional survey of ileal-anal pouch immune cells from ulcerative colitis patients. Gastroenterology 2021;160(5):1679–93. 33359089 10.1053/j.gastro.2020.12.030 PMC8327835

[35] Sinha SR Haileselassie Y Nguyen LP . Dysbiosis-induced secondary bile acid deficiency promotes intestinal inflammation. Cell Host Microbe 2020;27(4):659–70.e5. 32101703 10.1016/j.chom.2020.01.021 PMC8172352

[36] Maharshak N Cohen NA Reshef L . Alterations of enteric microbiota in patients with a normal ileal pouch are predictive of pouchitis. J Crohns Colitis 2017;11(3):314–20. 27613294 10.1093/ecco-jcc/jjw157

[37] Yanai H Ben-Shachar S Baram L . Gene expression alterations in ulcerative colitis patients after restorative proctocolectomy extend to the small bowel proximal to the pouch. Gut 2015;64(5):756–64. 24982202 10.1136/gutjnl-2014-307387

[38] Barnes EL Holubar SD Herfarth HH. Systematic review and meta-analysis of outcomes after ileal pouch-anal anastomosis in primary sclerosing cholangitis and ulcerative colitis. J Crohns Colitis 2021;15(8):1272–8. 33544128 10.1093/ecco-jcc/jjab025

[39] Penna C Dozois R Tremaine W . Pouchitis after ileal pouch-anal anastomosis for ulcerative colitis occurs with increased frequency in patients with associated primary sclerosing cholangitis. Gut 1996;38(2):234–9. 8801203 10.1136/gut.38.2.234 PMC1383029

[40] Quinn KP Urquhart SA Janssens LP . Primary sclerosing cholangitis-associated pouchitis: A distinct clinical phenotype. Clin Gastroenterol Hepatol 2022;20(5):e964–e973. 33549866 10.1016/j.cgh.2021.02.006

[41] Dubinsky V Reshef L Bar N . Predominantly antibiotic-resistant intestinal microbiome persists in patients with pouchitis who respond to antibiotic therapy. Gastroenterology 2020;158(3):610–24.e13. 31605691 10.1053/j.gastro.2019.10.001

[42] Shen B Kochhar GS Rubin DT . Treatment of pouchitis, Crohn's disease, cuffitis, and other inflammatory disorders of the pouch: Consensus guidelines from the International Ileal Pouch Consortium. Lancet Gastroenterol Hepatol 2022;7(1):69–95. 34774224 10.1016/S2468-1253(21)00214-4

[43] Shen B Achkar JP Lashner BA . A randomized clinical trial of ciprofloxacin and metronidazole to treat acute pouchitis. Inflamm Bowel Dis 2001;7(4):301–5. 11720319 10.1097/00054725-200111000-00004

[44] Barnes EL Agrawal M Syal G . AGA clinical practice guideline on the management of pouchitis and inflammatory pouch disorders. Gastroenterology 2024;166(1):59–85. 38128971 10.1053/j.gastro.2023.10.015 PMC11163976

